# Spatial variation and hot-spots of district level diarrhea incidences in Ghana: 2010–2014

**DOI:** 10.1186/s12889-017-4541-z

**Published:** 2017-07-03

**Authors:** Frank Badu Osei, Alfred Stein

**Affiliations:** 1 Department of Mathematics and Statistics, University of Energy and Natural Resources, Sunyani, Ghana; 2 Faculty of Geo-Information Science and Earth Observation (ITC), University of Twente, Enschede, Netherlands

## Abstract

**Background:**

Diarrhea is a public health menace, especially in developing countries. Knowledge of the biological and anthropogenic characteristics is abundant. However, little is known about its spatial patterns especially in developing countries like Ghana. This study aims to map and explore the spatial variation and hot-spots of district level diarrhea incidences in Ghana.

**Methods:**

Data on district level incidences of diarrhea from 2010 to 2014 were compiled together with population data. We mapped the relative risks using empirical Bayesian smoothing. The spatial scan statistics was used to detect and map spatial and space-time clusters. Logistic regression was used to explore the relationship between space-time clustering and urbanization strata, i.e. rural, peri-urban, and urban districts.

**Results:**

We observed substantial variation in the spatial distribution of the relative risk. There was evidence of significant spatial clusters with most of the excess incidences being long-term with only a few being emerging clusters. Space-time clustering was found to be more likely to occur in peri-urban districts than in rural and urban districts.

**Conclusion:**

This study has revealed that the excess incidences of diarrhea is spatially clustered with peri-urban districts showing the greatest risk of space-time clustering. More attention should therefore be paid to diarrhea in peri-urban districts. These findings also prompt public health officials to integrate disease mapping and cluster analyses in developing location specific interventions for reducing diarrhea.

## Introduction

Diarrhea is an ongoing public health threat, especially in developing countries. More than 1.7 billion episodes of diarrhea are recorded globally every year with the majority of these occurring in low and middle income countries [1–6]. Infection is mainly through contaminated water and food as a result of poor hygiene [7]. The persistence of diarrhea has been attributed to socio-economic inequalities such as low income levels, illiteracy, and inadequate safe water and sanitation [8–12].

In Ghana, diarrhea is the second most common health problem treated in out-patient departments. The nationwide reported diarrhea incidences increased from 725,976 cases in 2010 to 1,576,542 cases in 2014. Improvement in water and sanitation conditions still remains the long-term solution to reducing diarrhea. Under scarce budgetary resources, knowledge of the geographic hot-spots is a consequential alternative that could provide immediate solution with respect to decision making towards appropriate allocation of resources. Previous diarrhea studies in Ghana have predominantly focused either on single geographic units or the characteristics of the affected individuals [13–17]. These studies are unable to characterize the geographic areas of priority; hence a knowledge gap with respect to the geographical patterns still remains. Diarrhea morbidities vary across geographical areas; some areas are likely to sustain exceptionally high morbidities over time due to unplanned urbanization. The premise of this study also derives from previous population based studies [14, 15] that have suggested variation in diarrhea incidences at wider geographical units. Yet it is still unknown which areas have a higher than expected risk. It is thus imperative to identify areas of hot-spots as it is crucial to assist decision makers to assess programmatic needs, prioritize interventions and monitor progress. Children are the most vulnerable to diarrhea; knowledge of diarrhea hot-spots will also be an important step towards achieving the Sustainable Development Goal 3 (SDG 3) of ensuring healthy lives and promote well-being for all at all ages.

Our objective is to study the geographical patterns and hot-spots of diarrhea in Ghana. The demographic and socio-demographic indices amongst districts in Ghana are widely diverse as are diarrhea incidences. Since diarrhea morbidities are conditioned by socio-demographic factors, and since these factors are geographically correlated in space, we expect diarrhea morbidities to exhibit space-time clustering. For instance, unplanned rapid urbanization fueled by rural-urban migration can have substantial influence on diarrhea morbidities due to stress on existing amenities which do not meet the demands of the rising population. No previous study has explored the country-wide spatial patterns and hot-spots of diarrhea in Ghana. Our study is therefore focused on the spatial and space-time clustering of diarrhea. An additional purpose of the study is to examine the impact of urbanization on space-time clustering of diarrhea. Geographical hot-spots of diarrhea have been explored in Thailand [18] using the Local Indicator for Spatial Association (LISA) statistic. Kulldorff’s spatial scan statistic is well suited for detecting space-time clusters, hypotheses testing and making etiological inferences [19]. It has been used to study clustering of diarrhea in Ethiopia without formally recounting the possible causes of the clusters [20]. We recognize the challenge in the arbitrary selection of the maximum cluster size for spatial scan statistics [21]. We use the average behavior of spatial dependency structure, i.e. the practical range of the semi-variogram, to infer an empirical cluster window size. We consider a semi-variogram estimator that accounts for heterogeneous denominators of the rate parameter [22].

The remainder of the manuscripts is organized as follows. First, we develop empirical Bayesian smoothed maps of diarrhea. Second, we detect and map geographical areas of higher than expected incidences using the spatial scan statistics. Third, we describe the impact of urbanization on the occurrence of space-time clustering using logistic regression. We end with discussions and conclusions.

## Methods and analysis

### Study area and data

**Fig. 1:**
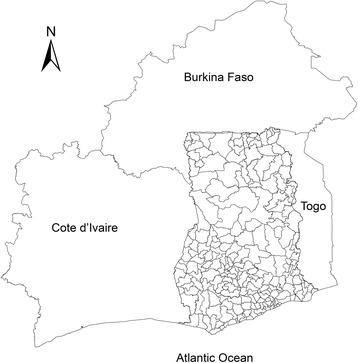
District map of Ghana showing the its neighboring countries; Cote d’Ivaire (left), Burkina Faso (top) and Togo (right)

Ghana is centrally located on the west coast of Africa (Fig. 1) with a total land area of 238,589 km^2^. It is a tropical region with varying temperatures and rainfall intensities. Ghana consists of ten administrative regions which are subdivided into 170 districts. Projections by the Ghana Statistical Service (GSS) puts the current population at 27,043,093. The spatial scale of our analysis is the district level of which data had been recorded. The population data were obtained from the Ghana Statistical Service (GSS). Diarrhea morbidities on outpatient records from 2010 to 2014 were obtained from the Centre for Health Information and Management (CHIM) of the Ghana Health Services (GHS).

### Mapping the spatial distribution of diarrhea risk

Area specific disease indices such as the relative risk, also called standard morbidity ratio (SMR), are important measures of neighborhood health status. The SMR is useful for guiding health interventions and allocations of health resources. In this study, we mapped the spatial distribution of the SMR rather than considering the disease rates in isolation. Let *O*_*i*_, *i* = 1 ,  …  , *m*, represent random variable of diarrhea cases *o*_1_ ,  …  , *o*_*m*_ in *m* districts. We assume that the *O*_*i*_are independently Poisson distributed *O*_*i*_ ~ *Pois*(*e*_*i*_ *r*_*i*_)with mean proportional to the unknown relative risk *r*_*i*_, such that $p\left({o}_i\right)=\left\{{\left({e}_i{r}_i\right)}^{O_i} \exp \left(-{e}_i{r}_i\right)\right\}/{o}_i!$, where *e*_*i*_is the expected number of cases in district *i*. Using the log-likelihood function $\log L={\sum}_{i=1}^n\left\{{o}_i \log \left({e}_i{r}_i\right)-{e}_i{r}_i\right\}$, the maximum likelihood estimator for the unknown relative risk is obtained as ${\widehat{r}}_i={o}_i/{e}_i$. The corresponding conditional mean and variance are $E\left[{\widehat{r}}_i\left|{r}_i\right.\right]={r}_i$ and $V\left[{\widehat{r}}_i\left| r\right.\right]={r}_i/{e}_i$, respectively. The expected number of cases*e*_*i*_ is defined in the absence of covariates as the number of cases in an epidemiologic “null model” of incidences *e*_*i*_ = *πn*_*i*_. Here, *n*_*i*_is the number of persons at risk in district *i*, and *π*is the individual level constant baseline risk estimated from the aggregated population by means of $\pi ={\sum}_{i=1}^m{o}_i/{\sum}_{i=1}^m{n}_i$. A major drawback of this estimate is that it leads to unstable estimates with areas of small populations showing the highest variability [23–25]. To account for this, we use the empirical Bayesian smoothing to borrow information across neighboring districts. This smoothing consists of obtaining a weighted average between the raw estimates for each district and the neighboring average, with weights proportional to the underlying population at risk [26]. In effect, districts with relatively small populations will tend to have their estimates adjusted considerably, whereas for districts with relatively large populations, the estimates will barely change. Following Clayton and Kaldor [26] and Gatrell and Bailly [27], the smoothed estimates of the relative risk is expressed as${r}_i^{\mathrm{EB}}={\varpi}_i{r}_i+\left(1-{\varpi}_i\right){\overline{r}}_i$, where the respective weights *ϖ*_*i*_for districts equal ${\varpi}_i={\sigma}_i^2/\left[{\sigma}_i^2+\left({\overline{r}}_i/{e}_i\right)\right]$. Here ${\overline{r}}_i$ and ${\widehat{\sigma}}_i^2$are the empirical local estimates of spatially varying prior mean and variance, respectively. We used the method of moments [25] to estimate ${\overline{r}}_i={\Sigma}_j{w}_{i j}{o}_i/{\Sigma}_j{w}_{i j}{e}_i$and ${\widehat{\sigma}}_i^2=\left[{\Sigma}_j{w}_{i j}{e}_i{\left({r}_i-{\overline{r}}_i\right)}^2\right]/{\Sigma}_j{w}_{i j}{e}_i-{\overline{r}}_i/\left({\Sigma}_j{w}_{i j}{e}_i/ n\right)$. We estimated the local mean ${\overline{r}}_i$ and the variance ${\widehat{\sigma}}_i^2$ based on the spatial neighborhood structure of the data*w*_*ij*_, such that *w*_*ij*_ = 1if districts *i* and *j* are neighbors, and zero otherwise.

### Spatial scan statistics

We used the spatial scan statistics developed by Kulldorff’s [21] to detect the presence of spatial and space-time clusters or hot-spots of diarrhea. We defined hot-spots as clusters with high than expected or elevated risk. The spatial scan statistic is a widely cluster detection tool to detect and evaluate geographical areas of excess risk against the null hypothesis of random distribution. It is based upon the principle that the number of cases in a geographic area follow a Poisson distribution according to a known underlying population at risk. This cluster detection method offers several advantages over other scan statistics methods (e.g. [28–30]): (1) it corrects for multiple comparisons, (2) it adjusts for the heterogeneous population densities amongst the different areas in the study, (3) it detects and identifies the location of the clusters without prior specification of their suspected location or size thereby overcoming pre-selection biases, and (4) it allows adjustment for covariates. The significance of Kulldorff’s scan statistic is widely acknowledged in spatial epidemiology [15, 19, 21, 31–41].

### Cluster window size

Critical to the spatial scan statistics is the selection of the maximum window size. Since there is no clear guideline for a choice, it is often chosen somewhat arbitrarily; e.g. as a percentage of the at risk population, or either based on experience or knowledge of the extent of clustering. Hjalmars et al. [34] suggest 10% of the population at risk whereas Kulldorff et al. [42] suggest 50% of the population at risk. Too large a window size may define too large areas as clusters which might be expensive and difficult for further epidemiological investigation. Larger sizes would also indicate areas of exceptionally low rates outside the window rather than areas of exceptionally high rates within the window. Too small a window may obscure important clusters. Since the maximum window size is related to the extent of spatial continuity, we estimated this using the semi-variogram. We assumed the relative risk as second order stationary random field; its theoretical semi-variogram *γ*(*h*)between any two districts *i* and *j* is *γ*(*h*) = 0.5*E*[*r*_*i*_ − *r*_*j*_]^2^, where*h* = |*i* − *j*|is the Euclidian distance between the centroids and *E* denotes the mathematical expectation. The corresponding method of moments (empirical) estimator [43], after forming multiple distance pairs, equals${\gamma}^{\ast }(h)=0.5{\left\{ N(h)\right\}}^{-1}{\sum_{i=1}^{N(h)}\left({r}_i-{r}_j\right)}^2$, where *N*(*h*)is the number of observation pairs separated by *h*. The traditional semi-variogram estimator, however, is not suited for the analysis of proportion since it does not account for heterogeneous denominators. Following Monestiez et al. [22, 44], the different pairs (*r*_*i*_ − *r*_*j*_)were weighted by their corresponding denominators $\frac{e_i\cdot {e}_j}{e_i+{e}_j}$ to homogenize their variance terms by dividing by weights proportional to their standard deviations. The adjusted experimental semi-variogram is then

$$ {\gamma}^{\ast }(h)=0.5{\left\{ N(h)\right\}}^{-1}{\sum}_{i=1}^{N(h)}\left\{\frac{e_i\cdot {e}_j}{e_i+{e}_j}{\left({r}_i-{r}_j\right)}^2-\overline{r}\right\} $$

where $N\left(\mathrm{h}\right)=\sum \frac{e_i\cdot {e}_j}{e_i+{e}_j}$ is a normalizing constant and $\overline{r}=\Sigma {e}_i\cdot {r}_i/\Sigma {e}_i$is an estimate of the weighted mean of *r*. Monestiez et al. [22, 44] developed the above semi-variogram to account for the spatially heterogeneous observation efforts of sparse animal sightings for mapping the relative abundance of species (*Balenoptera physalus*). Simulation studies indicated that this approach performs better than simple population-weighted approaches and Bayesian smoothers [45]. Permissible semi-variogram models by means of least squares were fitted to the experimental semi-variograms. From the fitted models, the largest range amongst the range parameters of the various models was noted as the maximum window size for the spatial scan statistics.

### Hot-spots detection

For the detection of purely spatial hot-spots, a circular window was defined which moves over the study region, centered on the centroid of each district. This varies from 0 to the maximum window size. This window size was defined based on the largest range of the semi-variogram models described in the previous section. Possible hot-spots are tested within the window whenever it centers on the centroid of each district. The null and alternative hypothesis are ${H}_0: r\left(\Omega \right)= r\left(\overline{\Omega}\right)$and ${H}_1: r\left(\Omega \right)> r\left(\overline{\Omega}\right)$, respectively, where *r*(Ω)and $r\left(\overline{\Omega}\right)$ are the relative risk within and outside the widows Ω and $\overline{\Omega}$. We can then express *o*(Ω) ~ *Pois*(*e*(Ω) ⋅ *r*(Ω)) and$o\left(\overline{\Omega}\right)\sim Pois\left( e\left(\overline{\Omega}\right)\cdot r\left(\overline{\Omega}\right)\right)$. Whenever the window finds a new case, the likelihood function for elevated risk within the window in comparison with those outside the window is calculated. The likelihood function for window Ω is proportional to.

$$ L\left(\Omega \right)=\begin{array}{c}\hfill \sup \hfill \\ {}\hfill \Omega \in \boldsymbol{\Omega} \hfill \end{array}{\left(\frac{o\left(\Omega \right)}{e\left(\Omega \right)}\right)}^{O\left(\Omega \right)}{\left(\frac{o\left(\overline{\Omega}\right)}{e\left(\overline{\Omega}\right)}\right)}^{O\left(\overline{\Omega}\right)}\times I\left(\frac{o\left(\;\Omega \right)}{e\left(\Omega \right)}>\frac{o\left(\overline{\Omega}\;\right)}{e\left(\overline{\Omega}\right)}\right) $$where *I*( )is the indicator function. The window Ω to be scanned by the spatial scan statistic is included in the set:**Ω** = {Ω_*ik*_|1 ≤ *i* ≤ *m*, 1 ≤ *k* ≤ *K*_*i*_}, where Ω_*ik*_, *k* = 1 ,  …  , *K*_*i*_, is the window composed of the (*k −* 1) nearest neighbors to district *i*. The window Ω^∗^ that attains the maximum likelihood is defined as the most likely hot-spot (MLH). We carried out the test of significance level by means of the Monte Carlo hypothesis testing [46]. We rejected the null hypothesis of no clustering when the simulated *p*-value is less than or equal to 0.05 for most likely hot-spots and 0.1 for secondary hot-spots [47].

For the detection of space-time hot-spots, a cylindrical window with a circular geographic base and height corresponding to time was used. The base of the cylinder is centered around one of several possible districts and its radius is varying continuously in size. The height of the cylinder reflects any possible time interval of less than or equal to half the total study period. The window then moves in space and time, visiting each time interval and geographic location [19, 21]. The likelihood ratio test statistic is constructed in the same way of the purely spatial hot-spots. However, the computational algorithm is in three rather than two dimensions [48]. Most likely hot-spots for different time lengths (i.e. 1, 2, 3, or 4-year length) were scanned.

### Odds of space-time hot-spots and population density

We applied binary logistic regression to unfold the odds of a particular district being a space-time hot-spot conditioned on the socio-demographic status. Here, we used urbanization*ρ*as the independent variable. Such variable is an invaluable proxy for many socio-demographic indicators known to influence diarrhea. For the observed value *y*, dichotomized as *y* = 1 if a district is a space-time cluster and *y* = 0otherwise, the conditional probability is $p\left( y=1\left|\rho \right.\right)=\frac{ \exp \left({\beta}_0+{\beta}_1\rho \right)}{1+ \exp \left({\beta}_0+{\beta}_1\rho \right)}$. This is linearized by means of the logit transform logit(*p*) = *β*_0_ + *β*_1_ *ρ*, where $\mathrm{logit}(p)= \log \left(\frac{p}{1- p}\right)$, *β*_0_is the intercept term, and *β*_1_is the fixed effect of the independent variable*ρ*. For meaningful interpretation and inferences, we classified urbanization into three strata, i.e. rural, peri-urban, and urban. Districts with predominantly rural communities were classified as rural (< 30% urban population), those with mixed urban and rural communities were classified as peri-urban (30%–70% of urban population), and those with predominantly urban communities were classified as urban (> 70% of urban population). We estimated three different fixed effect parameters for the odds ratios (OR),exp(*β*_*k*_) *k* = 1 , 2 , 3, corresponding to each stratum.

## Results and analysis

### Spatial distribution of relative risk

**Fig. 2:**
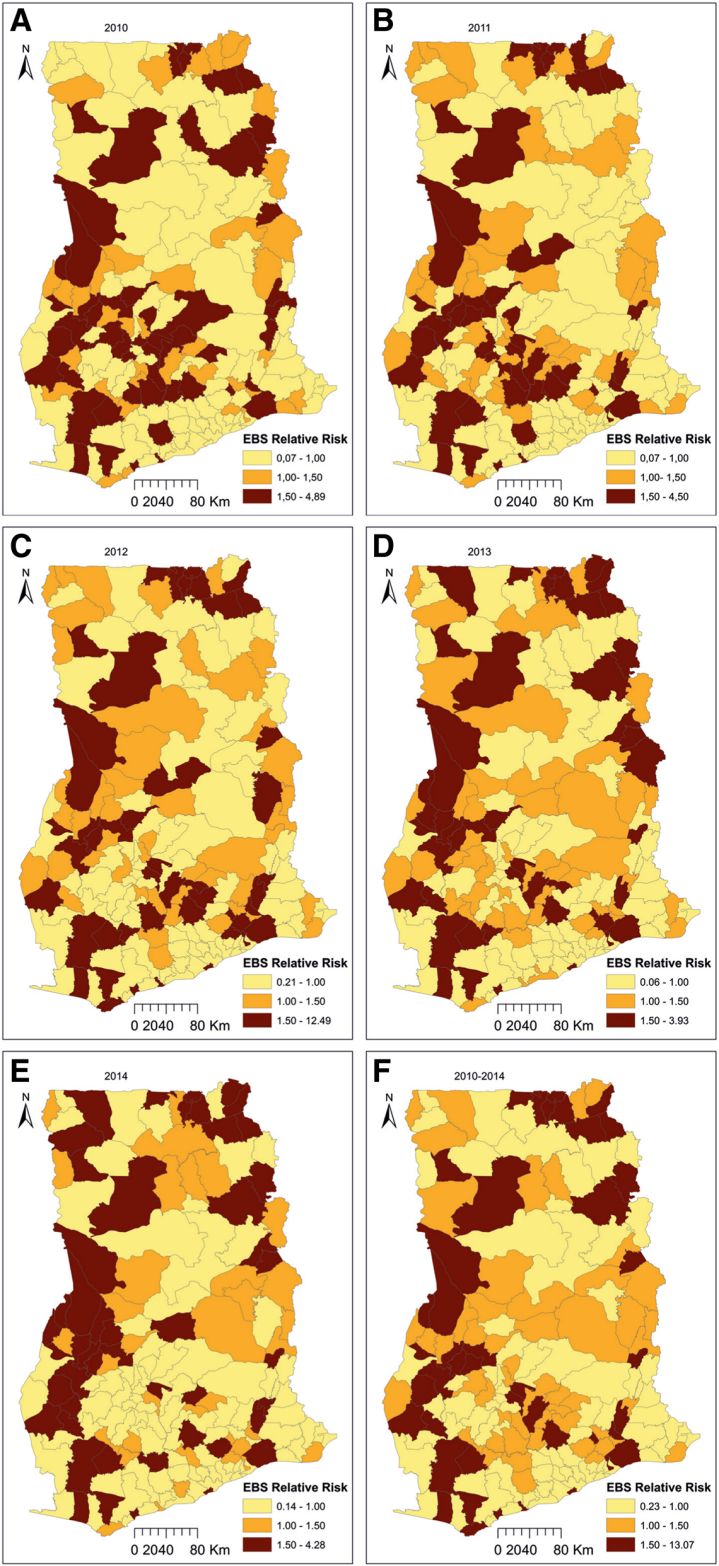
Spatial distributions of the empirical Bayesian smoothed estimates of the relative risks for 2010 (**a**), 2011 (**b**), 2012 (**c**), 2013 (**d**), 2014 (**e**), and cumulative estimates from 2010-2014 (**f**)

The overall risk of diarrhea varied with increasing trend ranging from 0.3% in 2010 to 0.58% in 2014. Seasonal variations were not analyzed due to the coarse temporal resolution of the data. Figure 2 shows the empirical Bayesian smoothed maps with remarkable spatial variations. We found temporal similarities of spatial patterns as some districts of either high or low rates remained same throughout the study period. Typically, the relative risk of districts within the mid-west parts remained pronounced and consistent throughout the study period.

## Spatial scan statistic

### Cluster window

**Fig. 3:**
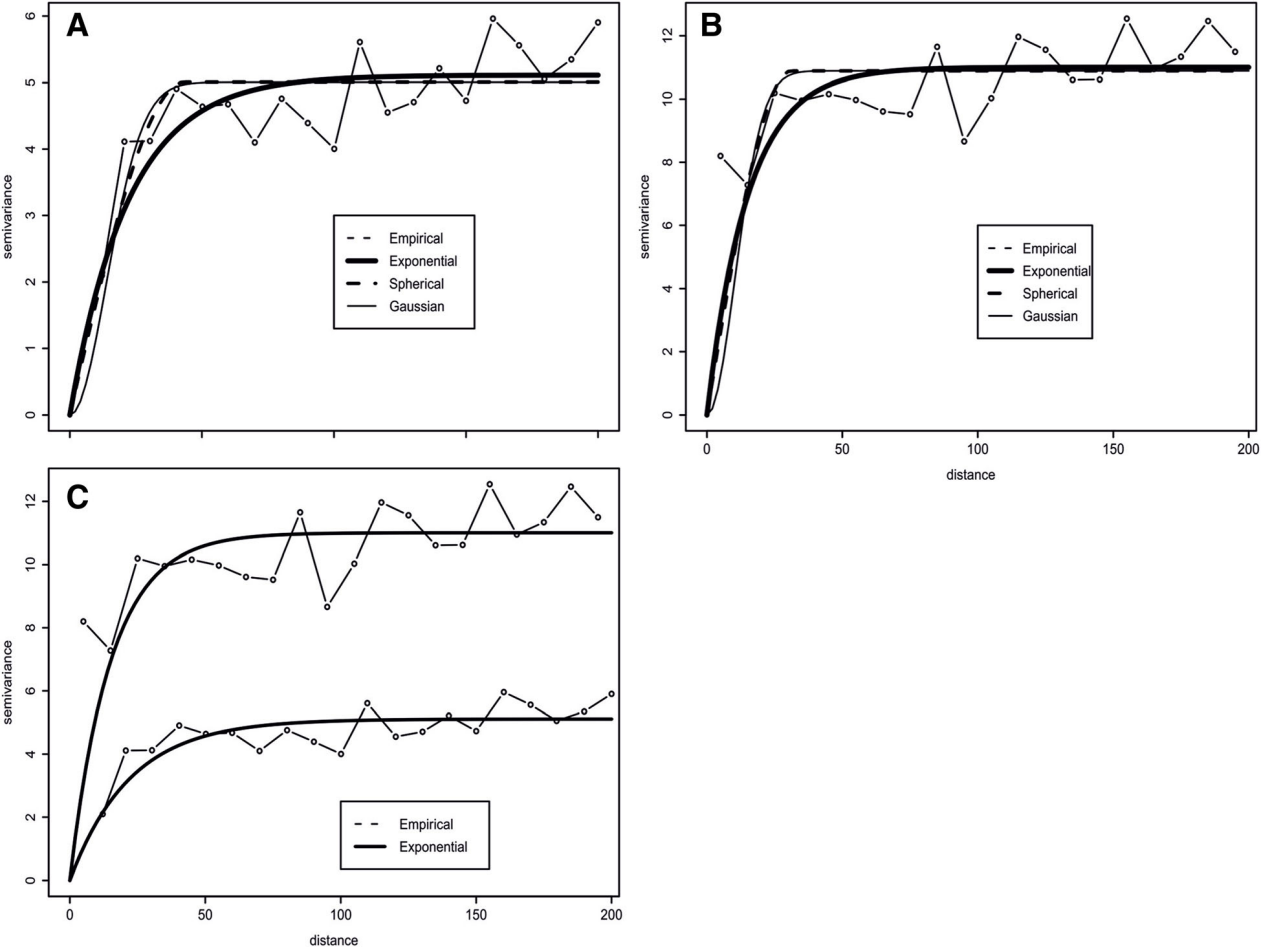
Empirical and theoretical semi-variogram models for both adjusted and traditional estimator. **a**: Adjusted semi-variogram estimator and theoretical models (exponential, spherical, and Gaussian). **b**: Traditional semi-variogram estimator and theoretical models (exponential, spherical, and Gaussian). **c**: Variations between exponential semi-variogram models estimated from the traditional and adjusted estimators

Setting the maximum window size was guided by the extent of spatial correlation of the relative risk. Experimental semi-variograms were computed using both traditional and adjusted estimators. The semi-variograms were estimated using 20 lags of 10 km. we fitted spherical, Gaussian, and exponential models using least squares. The exponential model expressed the largest range of spatial correlation, followed by the spherical and Gaussian models (See Table 1, Fig. 3a and b). When population heterogeneities were accounted for, the adjusted semi-variogram models had larger ranges and lower variances compared with the traditional estimators (Fig. 3c). Based on these, we chose a cluster window size of 70 km obtained from the practical range of exponential model of the adjusted semi-variogram estimator.

**Table 1:** Comparison between the adjusted and traditional semi-variogram models

	Adjusted		Traditional	
Model	Practical range (km)	Sill (%)	Practical Range (km)	Sill (%)
*Exponential*	70.00	5.11	45.29	11.01
*Spherical*	43.40	5.05	31.11	10.89
*Gaussian*	33.70	5.01	25.17	10.89

### Hot-spots detection

**Fig. 4:**
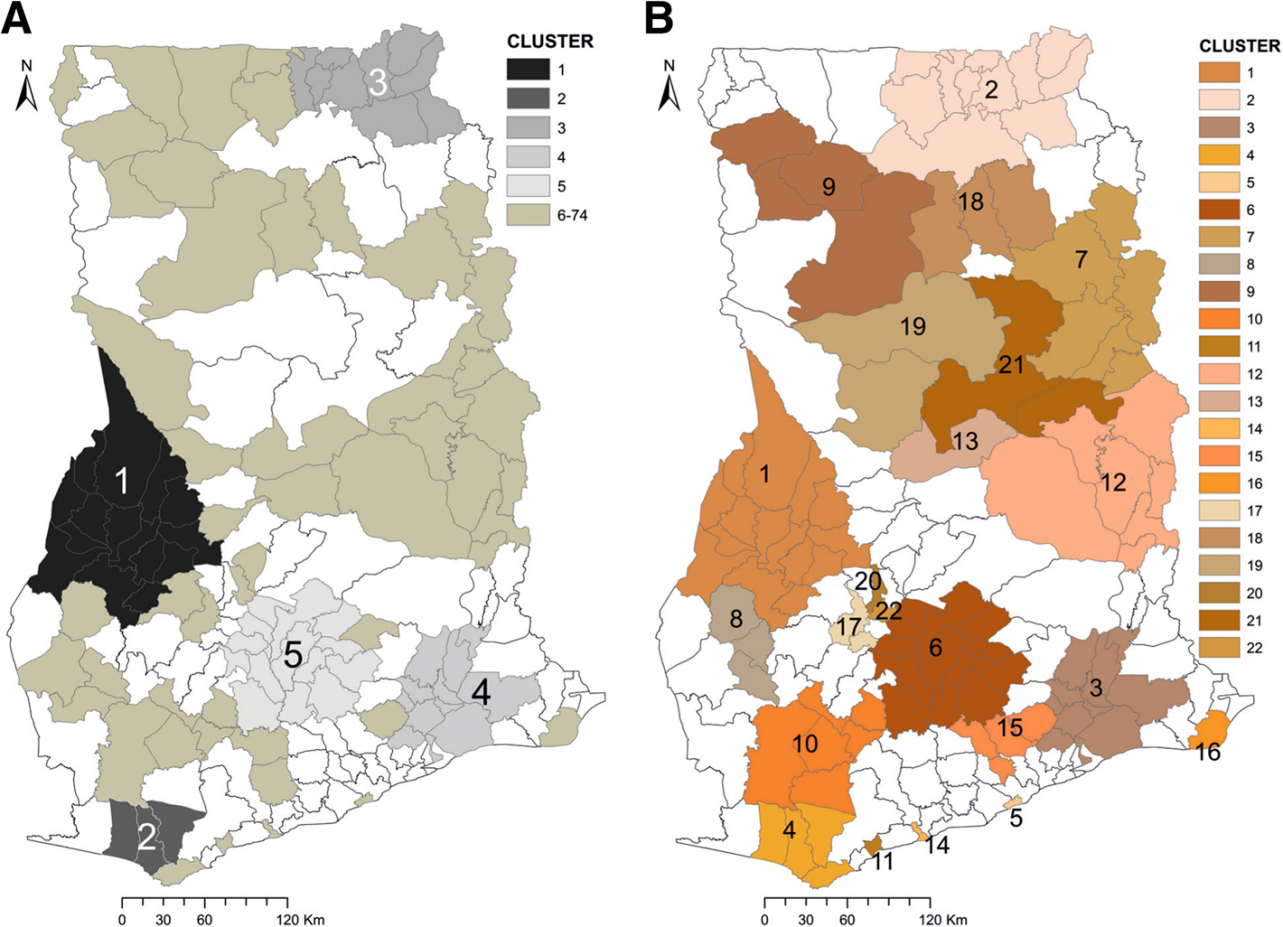
Spatial distribution of purely spatial (**a**) and space-time hot-spots (**b**) for diarrhea, 2010–2014

Statistically significant primary and secondary hot-spots were observed using the maximum window size of 70 km. The primary hot-spot encompassed 15 districts with higher than expected relative risk of 1.67 (*p* < 0.001). This hot-spot had 595,655 observed cases compared with 370,194.21 expected cases covering almost 6.03% of the population. A total of 73 statistically significant secondary hot-spots were also observed. Table 2 presents the characteristics of the first 5 spatial hot-spots of diarrhea, while Fig. 4a shows the spatial distribution of the spatial hot-spots.

**Table 2:** Characteristics of the first 5 spatial hot-spots of diarrhea, 2010–2014

Cluster	Radius	No. districts	LLR	*P*-value	Obs.	Exp.	RR
1	68,794.68	15	62,318.48	< 0.001	595,655	370,194.21	1.67
2	23,886.29	3	61,014.05	< 0.002	161,510	58,994.64	2.78
3	62,385.33	9	59,104.74	< 0.003	469,037	275,718.61	1.76
4	50,437.92	11	37,669.56	< 0.004	474,066	313,587.34	1.55
5	54,837.64	14	32,166.58	< 0.005	533,531	374,162.87	1.47

Statistically significant space-time hot-spots (*p* < 0.001) were also observed. This consisted of a primary hot-spot and 21 secondary hot-spots (Table 3). The primary hot-spot was observed in 2013–2014 encompassing 15 districts with a likelihood ratio of 71,867.76 and relative risk of 2.16 (Table 3, Fig. 4b). The first secondary hot-spot had similar characteristics as the primary hot-spot. This hot-spot occurred in 2013–2014 and encompassed 11 districts with a likelihood ratio of 54,964.05 and relative risk of 2.07. The existence of most of the space-time hot-spots spanned for more than one year and were considered as long-term hot-spots. Space-time hot-spots which existed for only one year period were considered as emerging hot-spots.

**Table 3:** Characteristics of the space-time hot-spots of diarrhea, 2010–2014

Cluster	Year	Type	No. Districts	LLR	*P*-value	Obs.	Exp.	RR
1	2013–2014	Long-term	15	71,867.76	< 0.001	319,714	152,454.13	2.16
2	2013–2014	Long-term	11	54,964.05	< 0.002	269,815	133,626.15	2.07
3	2012–2013	Long-term	11	41,221.36	< 0.003	240,573	126,969.68	1.93
4	2012–2013	Long-term	4	36,988.53	< 0.004	88,590	30,323.91	2.95
5	2013–2014	Long-term	1	29,235.56	< 0.005	36,094	7230.90	5.02
6	2012–2013	Long-term	14	27,769.41	< 0.006	250,430	151,480.90	1.68
7	2013–2014	Long-term	6	26,221.17	< 0.007	141,128	72,026.33	1.98
8	2013–2014	Long-term	3	25,288.21	< 0.008	56,661	18,544.33	3.07
9	2013–2014	Long-term	4	18,289.41	< 0.009	78,656	36,577.97	2.17
10	2013–2014	Long-term	6	13,295.16	< 0.010	120,375	72,578.63	1.67
11	2013–2014	Long-term	1	12,791.98	< 0.011	26,722	8326.13	3.22
12	2013–2014	Long-term	7	9924.87	< 0.012	107,572	67,950.10	1.59
13	2012	Emerging	1	6635.25	< 0.013	17,682	6435.55	2.75
14	2012–2013	Long-term	1	4412.37	< 0.014	31,200	17,428.19	1.79
15	2014	Emerging	4	3882.17	< 0.015	47,333	30,700.17	1.55
16	2013–2014	Long-term	1	1765.16	< 0.016	23,143	15,250.34	1.52
17	2010–2011	Long-term	2	1637.77	< 0.017	32,294	23,088.04	1.40
18	2014	Emerging	3	1315.55	< 0.018	24,646	17,455.64	1.41
19	2013–2014	Long-term	2	420.44	< 0.019	23,127	18,999.24	1.22
20	2014	Emerging	1	137.44	< 0.020	8609	7161.73	1.20
21	2014	Emerging	2	78.77	< 0.021	14,400	12,947.39	1.11
22	2011	Emerging	1	13.88	< 0.022	6031	5631.28	1.07

### Odds of space-time hot-spots and population density

From the results of the logistic regression model, the overall odds of space-time clustering was 1.62 (Table 4). The mostly likely stratum of space-time clustering is peri-urban districts. Space-time clustering is 11% higher in peri-urban districts (OR = 1.11; CI = [0.59–2.19]) than rural districts, and 43% lower in urban districts (OR = 0.57; CI = [0.23–1.39]) as compared with rural districts.

**Table 4:** Odds ratios and 95% confidence intervals of the logistic regression model

Variable	OR	2.5%	97.5%
*Intercept*	1.62	1.03	2.60
*Rural* (*reference*)	1		
*Peri-Urban*	1.11	0.56	2.19
*Urban*	0.57	0.23	1.39

## Discussion

This study aimed to explore and map the spatial variation and hot-spots of district level diarrhea incidences in Ghana. The findings showed temporal variation in the overall risk of diarrhea, with increasing burden since 2010 to 2014. This is probably due to unmatched population increase with the provision of safe sanitation and drinking water. From 2010 to 2014, Ghana’s population has grown from ≈24.6 to ≈27.2 million, a growth rate of ≈10.6%. This high population growth rate has caused major changes in socio-economic and demographic activities especially in rural and peri-urban districts where health and sanitation is already limited.

The empirical Bayesian smoothed maps show substantial variation in the spatial distribution of diarrhea with districts of higher/lower than expected risk clustered. This is a symptom of wider socio-economic inequalities amongst districts. We found diarrhea risk was more pronounced and consistent within the mid-west parts probably because these parts are dominated with semi-deciduous and rain forests. High precipitation, which is mostly associated with the semi-deciduous and rain forests has been found to exacerbate the risk of diarrhea infection [49–51]. Temporal similarities in the spatial patterns is also an indication of sustained transmission of diarrhea, suggesting that the spatial variation of the risk factors haven’t changed over the period. For instance, higher than expected risks were observed at the mid-west part of Ghana throughout 2010 to 2014 while the southern part continued to exhibit lower than expected risks. Complementarily, statistical inference of patterns using the spatial scan statistics detected both primary and secondary hot-spots, with the primary hot-spot (Cluster 1) detected within the mid-west part. This was the largest hot-spot with a radius of 68.79 km and encompassed 15 districts. We observed mutual occurrences between the empirical Bayesian smoothed maps and the hot-spots detected by the spatial scan statistics. The districts within the primary hot-spot also had higher than expected relative risks from the empirical Bayesian smoothed maps. Only few of the districts with higher than expected relative risk were not identified as hot-spots, thus indicating the significance of formal testing and inference in cluster analysis. While testing whether these spatial hot-spots were emerging or long-term, the space-time scan statistics recounted most of the spatial hot-spots as long-term (Fig. 4a and b). Specifically, the first five purely spatial hot-spots detected at the mid-west part of Ghana were also statistically significant long-term hot-spots. These clustering patterns imply less progress in prevention and control as well as unimproved hygiene and sanitation practices amongst in these districts. The epidemiological implication of the hot-spots can be deduced from the varying nature of the possible risk factors of diarrhea. Many known correlates of diarrhea are environmental and socio-demographic factors which are diversely distributed amongst the districts in Ghana. Since changes in population dynamics are highly variable in space [52], their effects on socio-demographic factors are also variable in space. Since such variation is spatially dependent and continuous, the expectation is that their ripple effects on health outcomes will also be spatially dependent and clustered. This implies that countermeasures should be opportunely undertaken, and focused on the areas of long-term hot-spots.

The impact of urbanization on space-time clustering was diverse amongst the various urbanization strata. Comparatively, space-time clustering was lowest in urban districts than rural and peri-urban districts. The underlying reason might be that the richer and better educated who are knowledgeable to prevent, and can secure safe water and sanitation for their households are mostly found in urban communities. Also urban populations have greater opportunities for health education and preventative health care. We found that space-time clustering was comparatively higher in peri-urban districts than in rural, which was inconsistent with our expectation. The reason might be that peri-urban districts are mostly transitional zones often neglected by urban planners; they are constantly under pressure by increasing populations from urban and rural population influx. For instance, the high cost of housing in urban districts restrains most rural*-*urban migrants and the urban poor to settle in peri-urban communities, thus heightening the creation of slums and informal settlements. Ghana has been able to achieve remarkable levels of access to improved drinking water in urban areas, yet meeting the needs of unserved and underserved communities as well as growing peri-urban areas is still a considerable challenge. As a consequence, such peri-urban settlements are often plagued with poor water and sanitation problems which are the well-known driving forces of diarrhea. We found no study linking rural-urban morphology to space-time clustering of diarrhea. This prompts that further studies are required to explore detailed comparative dynamics of diarrhea morbidities between the different urbanization strata.

The implications of our findings are stated with some caution. First, homogeneity in both population and disease counts are assumed. Thus, within-district variation is assumed to be absent to restrain our study to fall within the ecological analysis framework. While such studies are necessary for neighborhood health planning and large area intervention, they do not access and infer individual level risk characteristics, the so called *ecological fallacy*. Secondly, confounding and interaction effects have not been accounted for in this study. It is possible that rural-urban morphology would not matter if individual level variables mediating diarrhea risk were taken into account. Thirdly, this study used rural-urban morphology as the only proxy to capture socio-demographic risk of diarrhea. Studies have associated diarrhea with a mix of attributable socioeconomic inequalities such as low income level, illiteracy, inadequate water and sanitation [8–12]. Our future studies seek to explore the spatially varying effects of several of these factors on diarrhea morbidities. That notwithstanding, the overriding advantage of our findings is two- fold. First, this study shows the importance of spatial locations as a covariate in identifying and mapping areas of elevated and sustained transmission of diarrhea in Ghana. These maps provide valuable information to assist in appropriate allocation of health care resources for better control and prevention. Second, it divulges the dependency of high space-time clustering on peri-urban districts. This may provide a valuable factor for consideration in neighborhood health planning.

## Conclusions

This study has investigated the spatial variation of district level diarrhea incidences in Ghana by mapping and detecting hot-spots. Our study demonstrates the use of the extent of spatial continuity, the range parameter of the semi-variogram, to infer cluster window size for spatial scan statistics. We conclude that that the spatial distribution of diarrhea in Ghana is clustered, with evidence of emerging and long-term space-time hot-spots. The findings also infer that space-time clustering is higher in peri-urban districts compared with rural districts, and lowest in urban districts. These findings prompt health planners and policy makers to consider these patterns as critical when developing both short-term and long-term strategies to reduce diarrhea. We intend to further investigate risk factor characteristics of diarrhea within the emerging and long-term space-time hot-spots in the future.
